# Daily listening to Mozart reduces seizures in individuals with epilepsy: A randomized control study

**DOI:** 10.1002/epi4.12400

**Published:** 2020-05-27

**Authors:** Marjan Rafiee, Kramay Patel, David M. Groppe, Danielle M. Andrade, Eduard Bercovici, Esther Bui, Peter L. Carlen, Aylin Reid, Peter Tai, Donald Weaver, Richard Wennberg, Taufik A. Valiante

**Affiliations:** ^1^ Krembil Brain Institute Toronto ON Canada; ^2^ Institute of Biomaterials and Biomedical Engineering, and Electrical and Computer Engineering University of Toronto Toronto ON Canada; ^3^ Division of Neurology Department of Medicine University of Toronto Toronto ON Canada; ^4^ Department of Physiology University of Toronto Toronto ON Canada; ^5^ Division of Neurosurgery Department of Surgery University of Toronto Toronto ON Canada; ^6^ Department of Chemistry University of Toronto Toronto ON Canada; ^7^ Institute of Medical Science Faculty of Medicine University of Toronto Toronto ON Canada

**Keywords:** adjunctive therapies, epilepsy, music, neuromodulation, seizure reduction

## Abstract

**Objective:**

Epilepsy is one of the most common neurological disorders . Many individuals continue to have seizures despite medical and surgical treatments, suggesting adjunctive management strategies are required. Promising effects of daily listening to Mozart on reducing seizure frequency in individuals with epilepsy have been demonstrated over the last 20 years, but not in a rigorously controlled manner. In this study, we compared the effect on seizure frequency of daily listening to either Mozart K.448 or a spectrally similar, yet non‐rhythmic control piece. We hypothesized that there would be no difference in seizure counts when participants listened to Mozart K.448 vs when they listened to the control piece.

**Methods:**

We employed a randomized crossover design, in which each participant was exposed to both three months of daily listening to the first six minutes of Sonata for two pianos in D major by Mozart (Mozart K.448; treatment period) and three months of daily listening to phase‐scrambled version (control period). There was a three‐month baseline and a three‐month follow‐up period before and after the six‐month listening period, respectively. Change in seizure counts obtained from the seizure diaries was considered as the main study outcome.

**Results:**

Using three methodologies to investigate the existence of the treatment effect (paired *t* test, estimation statistics and plots, and Cohen's *d*), our results revealed a reduction in seizure counts during the treatment period, which was not observed for the control period (*P‐value* < .001).

**Significance:**

Using a spectrally similar control piece, our study advances previous reports that were limited by a “no music” control condition. Daily listening to Mozart K.448 was associated with reducing seizure frequency in adult individuals with epilepsy. These results suggest that daily Mozart listening may be considered as an adjunctive therapeutic option to reduce seizure burden in individuals with epilepsy.

**Key Point**

- Many individuals with epilepsy continue to have seizures despite medical and surgical treatments, suggesting a need for adjunctive management options.
- The promising effect of daily listening to Mozart in individuals with epilepsy has been reported for the past 20 years.
- The effect of daily exposure to Mozart K.448 (treatment piece) was compared with a phase‐scrambled version with no rhythmicity (control piece).
- Using a spectrally similar control piece, our study contrasted with previous reports that were limited by a “no music/sound” control condition.
- Our results revealed a reduction in seizures during the period of daily Mozart K.448 listening, an effect not observed for the control piece.

## 1 INTRODUCTION

Epilepsy is considered the most common serious neurological disorder in the world characterized by the recurrence of seizures, and affecting more than 50 million people worldwide.^1^ Many individuals continue to have seizures despite medical and surgical treatments, suggesting a need for adjunctive management strategies.

Previous neurophysiological and clinical studies have demonstrated that the use of different direct (such as electrical stimulation)^2^, ^3^ and indirect (such as music) stimuli to the brain may both evoke and prevent seizures.^4^, ^5^, ^6^, ^7^ Listening to music is likely the most complex rhythmic stimulus for modulating brain activity and has been associated with observation of different neurophysiological and neuropsychological changes in individuals with epilepsy and other conditions.^6^, ^8^, ^9^, ^10^, ^11^ In the 1990s, reports demonstrated beneficial effects on individuals’ spatial reasoning skills by listening to a particular Mozart piano piece (Sonata for two pianos in D major, also called Mozart K.448).^12^, ^13^ A landmark paper studying the effects of listening to Mozart K.448 in individuals with epilepsy demonstrated a reduction in interictal epileptiform discharges (IEDs), that were not observed during listening to a control old time Pop Piano tune.^14^ EEG recordings revealed that 23 out of 29 individuals demonstrated a significant reduction in the amount of IEDs during the time of exposure to Mozart K.448. Subsequently, a number of clinical studies in both adult and pediatric populations have explored the effects of listening to Mozart and have reported a reduction in IEDs and seizure frequency during the exposure time.^4^, ^9^, ^14^, ^15^, ^16^, ^17^, ^18^ As no specific auditory stimulus was used for the control periods in these studies, it remains unclear whether similar observations would follow in the event of using spectrally similar to Mozart K.448 yet rhythmically different auditory stimuli. Additionally, the need for randomized controlled studies on this topic has been discussed frequently in the literature^4^, ^6^, ^19^, ^20^ aiming to develop a better understanding of the reported effect.

In this work, we have explored the effects of daily listening to two different auditory stimuli on seizure frequency in adult individuals with epilepsy using a crossover study design, where each individual was exposed to both treatment and control auditory stimuli, serving as their own control. Mozart K.448 was chosen as the treatment piece**—**due to its ubiquity in the epilepsy literature.^4^, ^6^, ^14^, ^17^, ^21^ A phase‐scrambled version of the same piece containing similar frequency and amplitude content (albeit with no rhythmicity) was used as the control piece. Our hypothesis was that no difference in seizure frequency would be observed between the periods when individuals listened daily to the Mozart K.448, compared to when they listened daily to the phase‐scrambled version (control).

## 2 METHODS

### 2.1 Participants selection

The study protocol was approved by the University Health Network Research Ethics Board. Prior to starting the intervention, informed consent was obtained from all participants. The participants were chosen from a pool of adult individuals with epilepsy who were previously admitted to the Toronto Western Hospital (TWH) epilepsy monitoring unit and who had undergone either scalp or intracranial electroencephalography (EEG) recordings as part of a diagnostic of presurgical workup.^22^ Specific inclusion and exclusion criteria are presented in Table 1. In order to create a homogenous study population, we excluded individuals who had undergone resective epilepsy surgery, considering the contribution of different cortical areas in processing music.^6^, ^23^, ^24^, ^25^, ^26^

**TABLE 1:** Inclusion and exclusion criteria

Inclusion criteria	Individuals with epilepsy who are not satisfied with their current level of seizure control despite the use of various anti‐epileptic drugs (AEDs) Must have experienced at least three seizures during the baseline period, with the minimum number of one out of three seizures occurring within the last 2 months
Exclusion criteria	History of brain resection surgery (ie, epilepsy surgery) involving removal of any brain structure Any changes in an individual's AEDs during the one‐year intervention period Individuals receiving vagus nerve stimulation (VNS), deep brain stimulation (DBS), or following a ketogenic diet at the time of enrollment, or those planning to use such option(s) in the following year Individuals unable to recall their seizures; inability to document seizure occurrences in a diary during the one‐year intervention period, either by themselves or by their main caregiver Individuals unable to understand and speak English Individuals who score below 55% on the pitch perception and hearing impairment online test prior to starting the intervention^39^

### 2.2 Treatment and control stimuli

The treatment stimulus was the first six minutes and a half of the first movement of Mozart Sonata for two pianos in D major or Mozart K.448, performed by Alicia De Larrocha and André Previn. We chose the first movement of the sonata given the positive effects of the first movement^4^, ^14^ and its practical length to encourage compliance with the study. Our choice of control piece was motivated by the hypothesis that Mozart K.448 contains periodicities that might underlie its potential therapeutic effects.^27^ We were also motivated by the objective that the control piece should be spectrally similar (ie, the power spectrum should be similar) to avoid the confound of using both a spectrally and rhythmically dissimilar control piece. Thus in order to preserve the spectral contents of Mozart K.448 while destroying its rhythmicity, we utilized a common approach to generating surrogate time series data that disrupts long‐term correlated structure by shuffling the phases of the different frequency components of the original piece.^28^ A phase‐scrambled version of the treatment piece was created by replacing phase information of the complex Fourier transform of the Mozart K.448 piece with a random number between –π and π while preserving the power of each frequency component and then computing the inverse Fourier transform as described previously in the literature.^29^ Subsequently, the final control piece sounded like noise with no rhythmicity. The treatment and control stimuli were referred to as Mozart and scrambled Mozart to the participants during this study.

### 2.3 General design

The randomized control research study employed a crossover design by using a computer‐generated algorithm written in MATLAB^©^ to assign participants to two groups. The individuals in group A started the intervention by listening to the treatment stimulus—Mozart K.448—once a day for a total period of three months (treatment period) and switched to listening to the control piece once a day for the following three months (control period). Individuals in group B engaged in identical listening regimens. However, they started with the control piece, followed by Mozart K.448. Additionally, there were three‐month baseline and three‐month follow‐up periods before and after the six‐month listening period. The information regarding participants previous musical history is presented in Figure S2. During the intervention period, participants were given the option of listening to the sound stimuli by either logging into their personal accounts on a website specifically designated for this study or by using the electronic copy of the sound files in a.wav format. They could set the volume to their comfort level and could choose freely the timing of the intervention each day, with no specific instruction on the use of earphones or speakers.

### 2.4 Data collection and the main study outcome

Since not all of the individuals with epilepsy were able to classify and differentiate seizure types and auras using common scientific terms, we instructed the participants to discuss with us the different and personal ways they classify their seizure type(s) using their own words. The participants were then advised to record their seizures in their seizure diaries, using their own symbols to label them differently, notably in the event of auras or multiple types of seizures. Adding the participants self‐reported diary next to the existing notes from their neurologist and the previously available history of the EEG findings describing their seizure type(s), the seizure diary entries served as the primary outcome of the study. During the study visits to the hospital, seizure diaries were obtained from participants after each three‐month period (baseline, treatment/control, and follow‐up) (see Table S1 for complete description of study visits and collected data). In order to estimate the compliance rate, the participants were provided with a username and password, to gain access to our designated website to listen to the auditory stimuli (Mozart K.448 or the scrambled version of it). Individuals were asked to answer a survey question after finishing their daily listening. If the total time spent on the website (calculated as the time of survey submission minus the time of logging into the website) was equivalent to the duration of the auditory stimulus, it was presumed that the individual followed the intervention on that day. The participants who did not choose to use the website for this purpose were asked to mark the days they skipped listening to the auditory stimulus in the seizure diary provided to them. The compliance rate for each period was estimated as the number of days that an individual listened to a stimulus over the total length of each period in days.

### 2.5 Statistical analysis

Statistical analysis was performed following both an intention to treat (ITT) and per‐protocol (PP) analysis. For the ITT analysis, missing observations due to dropouts were handled by the last observation carried forward (LOCF) method, using the last observation of an individual's seizure counts before dropout of the rest of the intervention. The total numbers of seizures during the treatment and control periods were considered as paired observations for each individual and were normalized with respect to the individual's total number of seizures during the baseline period. Shapiro‐Wilk test was used to check the normality of the dataset, using the Python statistical functions module.

The effect size was analyzed using three different methods: the paired Student's *t* test considering the normal distribution of the dataset,^30^ estimation statistics and plots, and Cohen's *d*. In addition to providing the *P*‐value, we also focused on the magnitude of the observed effect size and its’ precision using estimation statistics and plots,^31^ following the methodology suggested by Ho and colleagues.^32^ In the estimation plots, the difference‐axis origin was aligned with the mean of the treatment phase, relating the observed values of the total number of seizures during the treatment and control periods to their mean.^32^ The mean differences between paired observations were used to calculate the effects size with 95% bootstrapped confidence interval (95%CI) and presented in the following format: “*mean [95% CI lower bond, upper bond]*”.^32^ All of the statistical tests and calculations were carried out using the DABEST Python package.^32^

## 3 RESULTS

**FIGURE 1:**
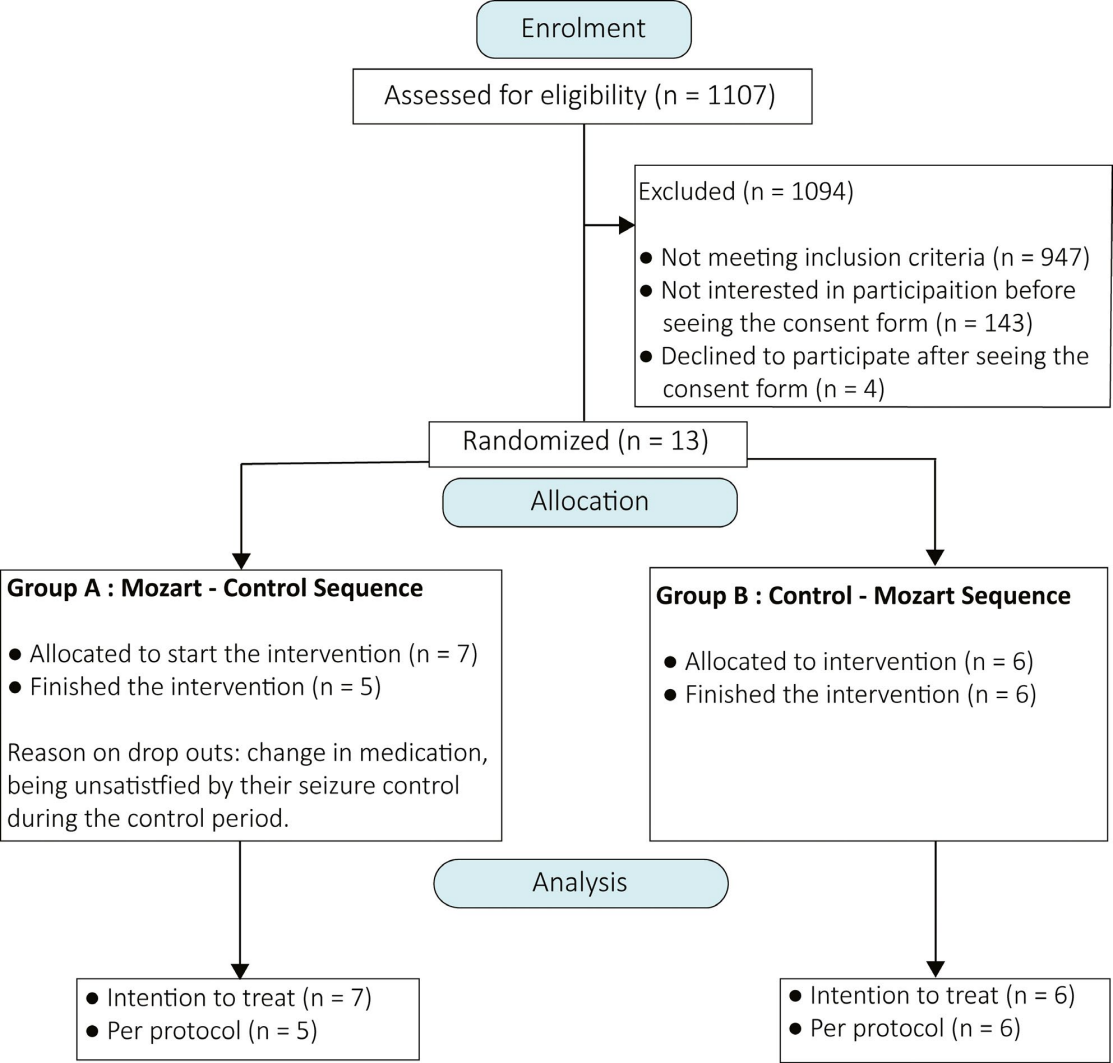
Participants flow diagram

The study group involved 13 adult individuals with epilepsy (eight female and five male), between 26 and 75 years of age with a mean age of 45.8 years, and an average of 2.3 AEDs per individual (Figure 1). The demographic information of the participants is summarized in Table 2. The compliance rate was estimated as 83 ± 11% and 72 ± 16% during the treatment and control periods, respectively (n = 11 after excluding two dropouts, see below). Spearman's correlation computation revealed no correlation between the seizure counts and the compliance rate among individuals during the treatment period (n = 11, *r*_s_ = .155, *P‐value* = .15) or during the control period (n = 11, *r*_s_ = −.08, *P‐value* = .81).

**TABLE 2:** Participants demographic information

Study ID	Gender	Age	AED(s)	MRI Findings	Seizure Type
01	F	75	AC, LZ	Normal	A
02	F	50	CB	Left polymicrogyria	CP, N
03	F	56	LC, LV	Right mesial temporal sclerosis	CP, GTC
04	M	29	BR, LC, LM, OX	Normal	N
05	F	26	CB, LM, LC, CL	Normal	SP, CP, N
06	M	32	LV, CB, VA, CL	Generalized brain atrophy	CP, GTC
07	M	33	LM	Right temporal encephalocele	CP
08	M	28	PH, LC, LZ	Occipital horn dilatation	CP, GTC
09	F	42	LM, LC	Normal	CP
10	F	53	CB, LV	Normal	CP, GTC
11	M	67	CB, LC, PR	Normal	CP
12	F	42	TO	Multiple bilateral white matter hyperintensities	CP
13	F	63	VA	Normal	CP

Abbreviations: A, absence; AC, acetazolamide; BR, brivaracetam; CB, carbamazepine; CL, clobazam; CP, complex partial; GTC, generalized tonic‐clonic; LC, lacosamide; LM, lamotrigine; LV, levetiracetam; LZ, lorazepam; N, nocturnal; OX, oxcarbazepine; PH, phenytoin; PR, perampanel; SP, simple partial; TO, topiramate; VA, valproic acid.

Two participants (P08 and P10) withdrew from the study after finishing the treatment period and during the control period; both belonged to group A (treatment‐control sequence). One individual from group A became seizure‐free during the treatment period (P12**—**Figure S1). All the participants in group B (control‐treatment sequence) showed a lower mean number of seizures during the treatment period compared to their baseline period except one (P09**—**Figure S1). The seizure diary counts for all participants during the intervention time can be found in the Figure S1.

**FIGURE 2:**
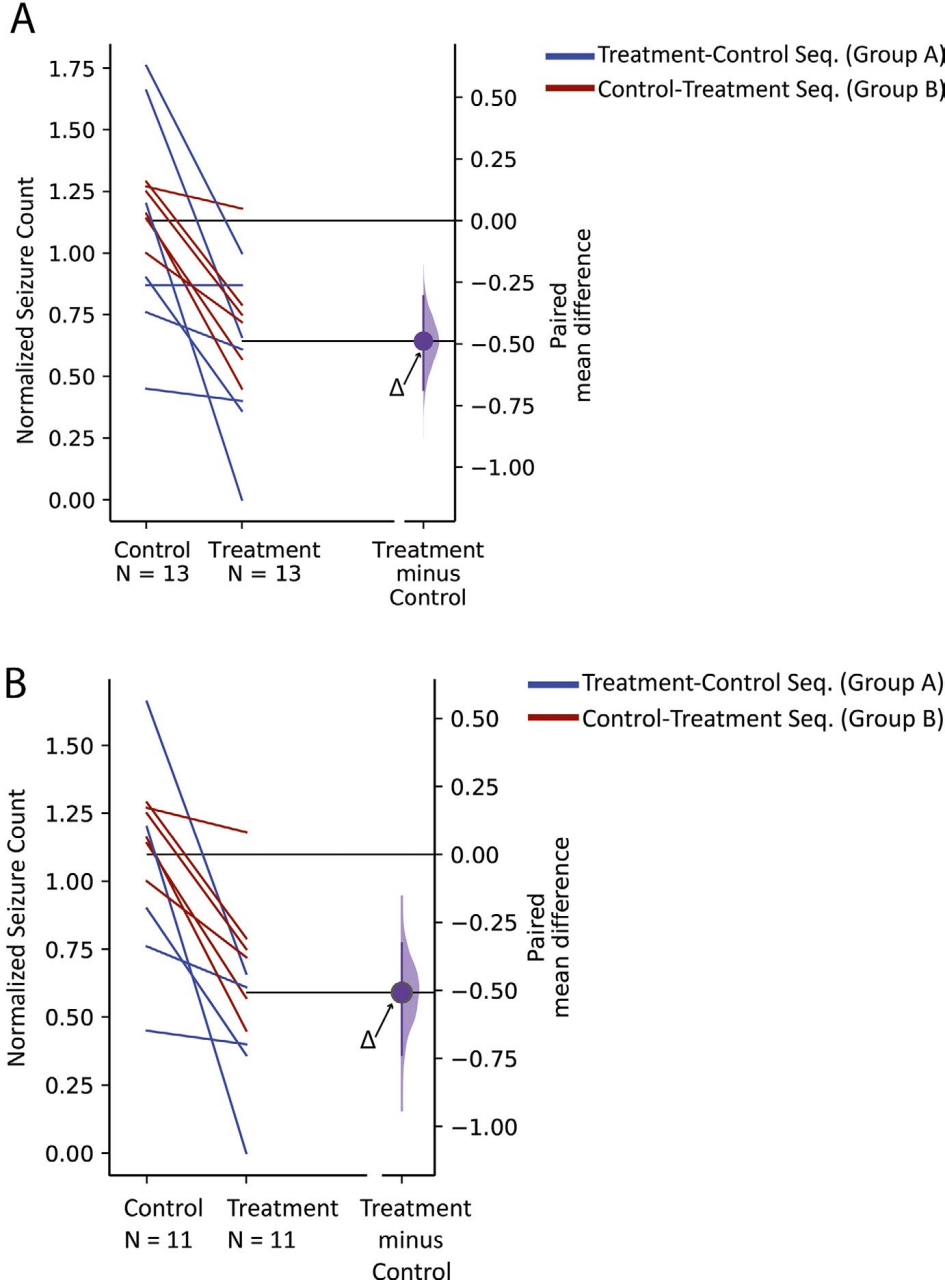
Estimation graphics to display the effect size under (A) intention to treat (n = 13) and (B) per‐protocol (n = 11) analysis. The blue and red lines connect the paired observations on the seizure counts during the control and treatment periods for individuals in groups A and B, respectively. Horizontally aligned with the mean of the treatment group, the purple curve represents the resampled distribution of the mean differences between the seizure counts during the control and treatment periods or Δ, indicated by the purple circle. The axis origin, indicated by a black horizontal line, by definition represents the mean of the null hypothesis and is equal to zero

Statistical analysis revealed the existence of a treatment effect. Under both ITT and PP analysis, individuals in group A (treatment‐control sequence) showed slightly larger treatment effect (ie, larger difference in seizure counts between treatment and control groups) than those in group B (control‐treatment sequence). For individuals in both groups A and B, the mean seizure count was reduced during the treatment period as compared to the control period under ITT (−44% for group A, −25% for group B) and PP analysis (−59% for group A, −25% for group B). The results of paired Student's *t* test and the estimation plots (Table 3 and Figure 2) confirmed the existence of a treatment effect under both ITT (*P‐value* = .0005, *t‐value* = 4.75, Figure 2A) and PP analysis (*P‐value* = .0009, *t‐value* = 4.61, Figure 2B). Using the mean of the normalized seizure counts during the treatment and control periods, paired Cohen's *d* was calculated as 1.5 [95% CI 0.7, 2.1] and 1.6 [95% CI 0.7, 2.5] under ITT and PP analysis, respectively. Cohen's *d* values are compatible with a large effect size in the study (Cohen's *d* > 0.8 was considered as large effect size by definition). The post‐listening effect of listening to the treatment piece was addressed during the follow‐up periods. Individuals in groups A (control‐treatment sequence) and B (treatment‐control sequence) showed 18% and 24% increase on their average normalized seizure counts during the follow‐up period, respectively (normalized by the seizure counts during the last three‐month before the follow‐up period). Individuals with normal and abnormal MRI demonstrated no significant difference between their seizure counts (Student's *t* test, *P‐value* = .3, *t‐value* = −0.9), suggesting both groups of patients benefit similarly from listening to Mozart. In summary, individuals demonstrated a 35% reduction in their seizure counts on average while listening daily to Mozart K.448 for three months compared to daily listening to a spectrally identical (non‐rhythmic) control piece.

**TABLE 3:** Results of Student's *t* test for paired samples and the related statistical analysis

	Intention to Treat			Per‐Protocol		
	Period			Period		
	Treatment	Control	Within‐individual differences (Treatment‐Control)	Treatment	Control	Within‐individual differences (Treatment‐Control)
Mozart‐Control						
Mean (SD)	0.6 (0.3)	1.2 (0.6)	−0.6 [−1.2 to −0.1]	0.4 (0.2)	1 (0.4)	−0.5 [−1.0 to −0.1]
Sample size	7	7	7	5	5	
Control‐Mozart						
Mean (SD)	0.7 (0.2)	1.2 (0.1)	−0.4 [−0.7 to −0.2]	0.7 (0.2)	1.1 (0.1)	−0.4 [−0.6 to −0.2]
Sample size	6	6	6	6	6	
Treatment Effect						
Mean (SD)	‐	‐	−0.5 [−0.7 to −0.3]	‐	‐	−0.5 [−0.7 to −0.3]
*t* test for paired sample	‐	‐	*P*‐value = 0.0005 *t*‐value = 4.75			*P*‐value = 0.0009 *t*‐value = 4.61
Sample size	13	13		11	11	

Note

The mean values correspond to the mean of the normalized total number of seizures in each three months period. The numbers shown for within‐individual differences correspond to the mean [95% CI].

## 4 DISCUSSION

In this study, we investigated the effects of daily listening to two different auditory stimuli on seizure counts in adult individuals with epilepsy. We employed the first 6 minutes of Mozart K.448 as our treatment piece and a control stimulus with the same power spectrum to that of the Mozart piece, but shuffled in phase, rendering it non‐rhythmic. We hypothesized that there would be no difference in individual seizure counts during the period of daily listening to the treatment stimulus in comparison with the period of daily listening to the control stimulus. Using different methodologies to estimate effects size, our results revealed a reduction in seizure counts during the period of daily listening to Mozart, which was not observed for the control stimulus (Table 3, Figure 2). All participants except one (P09, from group B) exhibited a reduction in their seizure counts during the treatment period in contrast to their baseline period (Figure S1). One participant (P12) became seizure‐free during the three months of daily listening to Mozart K.448 (Figure S1).

Additionally, by estimating the compliance rate during the treatment and control periods, we assessed how scrupulously our participants followed the intervention. The high compliance rate during both control and treatment periods (ie, not just during the treatment period) is notable. No information was provided to the participants during their enrollment in the study to bias or direct them toward believing which stimulus might be more likely to have a positive effect. However, it should be noted that the potential positive effects of listening to Mozart have been mentioned in the media over the past 15 years and it would be naive to assume that participants were necessarily blinded to the effects of the employed auditory stimuli not only in this study, but also as a burgeoning topic for other studies.

We found the high compliance rates during both treatment and control periods to support the findings of a recent survey among 40 individuals with epilepsy in the United States, which revealed that 75% of participants were interested in listening to specific music on their mobile phones in order to reduce their seizure frequency.^33^

There are many environmental factors that could also affect seizure counts in individuals with epilepsy, such as lack of sleep and varying levels of both physical and mental stress. Considering the length of our intervention, we cannot comment on how changes in such environmental factors may have impacted the observed effect across our participants. Our results are similar to those reported by Hughes and colleagues,^14^ who observed positive effects on reducing IEDs in individuals with epilepsy listening to Mozart K.448. The control pieces used in both our study and the study from Hughes and colleagues^14^ were distinctive from the Mozart piece by containing a different complex rhythmic structure, with our control piece containing no rhythmicity at all. Our findings, in addition to the results reported by Hughes and colleagues ^14^, ^27^, highlight the potential importance of the rhythmic structure of the musical pieces on seizure activity in individuals with epilepsy; however, this remains to be further explored. In the pediatric population, recent reviews and meta‐analyses have revealed that despite initial promising results, the effectiveness of listening to Mozart on reducing seizure frequency in the pediatric population has yet to be determined, a conclusion attributed to limitation in study quality and lack of sufficient data sharing.^19^

Our study design and protocol differed from previous clinical studies, which primarily used a parallel design^4^, ^16^, ^21^, ^34^ except for one which used a crossover design.^18^ None of these studies used a control piece during the intervention. Additionally, previous studies used a wide range of treatment periods ranging from three months of listening to Mozart K.448 to up to a year.^19^, ^35^ Despite the use of different protocols, previous work reported the existence of a reduction of seizures while listening to Mozart.

In terms of the existence of a general treatment effect during the period of listening to Mozart K.448, we found our results in agreement with the only other randomized control study available on adult individuals with epilepsy—which involved 36 individuals—notwithstanding a number of differences between the two protocols used and the intervention design.^4^ We were unable to directly compare the changes in seizure counts during the control period in our study to the reported observations from Bodner and colleagues’ control group^4^ since our study featured a control auditory stimulus. Our results under PP analysis demonstrated an increase in normalized seizure counts for all participants during the daily exposure to the control piece. In Bodner and colleagues’ study,^4^ however, with no auditory exposure, over half of the individuals in the control group exhibited either no change or an increase in their seizures.

All of our participants, except the two outliers from groups A and B (P12 and P09 in Figure S1), showed similar increase in their average seizure counts after terminating their exposure to both the treatment and control stimuli and during the follow‐up period (Figure S1). This is in contrast with the reported follow‐up results by Bodner and colleagues^4^ who suggested that the effects of an extended one‐year treatment can result in long‐term reduction of seizure rates in the majority of individuals in the treatment group. We speculate that there could be a number of factors playing a role in this observed difference. First, by asking individuals to listen to an auditory stimulus once a day, we tried to intentionally design a study that would be practical to follow within the usual day to day setting of the life of an adult individual with epilepsy. In contrast, Bodner and colleagues’ study involved playing Mozart K.448 at regular intervals during the individuals’ sleep (10 hours nightly) using the central sound system in a long‐term facility. Second, the length of our intervention period was comparatively much shorter due to feasibility reasons (3 months vs 12 months, respectively), and we speculate that this factor could have played a role in the observed difference. We have yet to determine whether the positive treatment effects could persist through longer treatment periods after terminating the treatment phase.

The exact neural mechanisms underlying reducing seizures by listening to music have not been determined yet. The potential theories on the effect of listening to music on central nervous system have been discussed in detail in a number of the previous review work.^6^, ^20^, ^36^ A number of studies additionally have suggested a potential mechanism involving activation of different cortical areas while listening to music.^4^, ^14^, ^27^, ^37^ Interestingly, this effect was reported to be missing in using other musical pieces. The existence of auditory stimulation of the brain while listening to this piece could be related to the presence of the unique long‐term periodicities in Mozart K.448.^14^, ^27^

As with other experimental work, this study comes with its own limitations. Considering the size of our study population and that variability exists between individuals, our study might have benefitted from a larger sample size. However, our use of a crossover design permitted us to draw statistical inferences from a smaller sample size than would be required for a parallel design while achieving the same type I and type II errors.^37^, ^38^ During the enrollment phase of our study, only 13 out of 147 potential participants ultimately joined the study. This might suggest the existence of a placebo effect, especially considering the high compliance rate observed during both treatment and control periods. The positive effects of listening to Mozart on reducing seizure counts have been previously reported to be generalized across genders and seizure types (focal, generalized, focal and generalized, and generalized and myoclonic), with a greater reduction in seizures among adult individuals with idiopathic epilepsy.^4^ In this work, we carried out the randomization independent of the individuals’ seizure types, gender, age, and baseline seizure activity. This choice was done in order to increase the enrollment rate, considering the wide exclusion criteria we used.

One of the most significant challenges of studies with crossover designs is the existence of the carry‐over effect.^37^ Depending on the feasibility factors, such as the study duration and financial limitations, the conventional way to address this in the pharmacology literature is by considering a washout window between different periods of the intervention, with its length determined by an individuals pharmacokinetic and pharmacological responses.^37^ However, the main difficulty with adding a washout period is that one can hardly be sure that it actually works.^37^ In our study, we used two different sound stimuli as the treatment and control agents. However, with no information to guide the length of a carry‐over effect in the music literature, along with the need to increase the number of study visits for participants, we did not include a washout period between the treatment and control periods in our study. As discussed before, in comparison with observations during the follow‐up period to Bodner and colleagues’ work,^4^ we suggest further studies are needed to fully investigate whether the observed treatment effects only exist during the time individuals are actively participating in such interventions or not. In summary, to the best of our knowledge, this is the first randomized control study in adult individuals with epilepsy using a crossover design, which attempts to answer the question of the effect of listening to Mozart^14^ on seizure control. Our results revealed a reduction in seizures through daily listening to Mozart K.448 in adult individuals with epilepsy. Exploring the potential mechanism behind the observed effect, in addition to designing studies that include a control stimulus containing rhythmicity, are some of the suggested topics to be investigated in future works. We hope that the combination of our study design, data and statistical results, and the observed promising effect can serve as a foundation for future work on this topic.

## CONFLICT OF INTEREST

None of the authors have any conflict of interest to disclose. We confirm that we have read the Journal's position on issues involved in ethical publication and affirm that this report is consistent with those guidelines.

## Supporting information

Supplementary Material

## References

[1] Reynolds EH . The ILAE/IBE/WHO epilepsy global campaign history. Epilepsia. 2002;43:9–11. 10.1046/j.1528-1157.43.s.6.5.x 12190968

[2] Penfield W , Jasper H . Epilepsy and the functional anatomy of the human brain. South Med J. 1954;47(7):704.

[3] Bauer S , Baier H , Baumgartner C , Bohlmann K , Fauser S , Graf W , et al. Transcutaneous vagus nerve stimulation (tVNS) for treatment of drug‐resistant epilepsy: a randomized, double‐blind clinical trial (cMPsE02). Brain Stimul. 2016;9(3):356–63. 27033012 10.1016/j.brs.2015.11.003

[4] Bodner M , Turner RP , Schwacke J , Bowers C , Norment C . Reduction of seizure occurrence from exposure to auditory stimulation in individuals with neurological handicaps: a randomized controlled trial. PLoS ONE. 2012;7(10):e45303–e45310. 23071510 10.1371/journal.pone.0045303 PMC3469625

[5] Critchley M . Musicogenic epilepsy In: Critchley M., Henson RA, editors. Music and the Brain(pp. 344‐353). Oxford, UK: Butterworth‐Heinemann; 1977: 344–53.

[6] Maguire MJ . Music and epilepsy: a critical review. Epilepsia. 2012;53(6):947–61. 22612325 10.1111/j.1528-1167.2012.03523.x

[7] Stern J . Musicogenic epilepsy In: Aminoff MJ, Boller F, Swaab DF, editors. Handbook of Clinical Neurology (Vol. 129, pp. 469‐477). Amsterdam: Elsevier; 2015:469–77. 10.1016/B978-0-444-62630-1.00026-3 25726285

[8] Smith CA , Morris LW . Differential effects of stimulative and sedative music on anxiety, concentration, and performance. Psychol Rep. 1977;41(3_suppl):1047–53. 601132 10.2466/pr0.1977.41.3f.1047

[9] Turner RP . The acute effect of music on interictal epileptiform discharges. Epilepsy Behav. 2004;5(5):662–8. 15380117 10.1016/j.yebeh.2004.07.003

[10] Koelsch S . A neuroscientific perspective on music therapy. Ann N Y Acad Sci. 2009;1169(1):374–84. 19673812 10.1111/j.1749-6632.2009.04592.x

[11] Fachner J , Gold C , Erkkilä J . Music therapy modulates fronto‐temporal activity in rest‐EEG in depressed clients. Brain Topogr. 2013;26(2):338–54. 22983820 10.1007/s10548-012-0254-x

[12] Rauscher F , Shaw G , Ky K . Mozart and spatial reasoning. Nature. 1993;365:611. 10.1038/365611a0 8413624

[13] Rauscher FH , Shaw GL , Ky KN . Listening to Mozart enhances spatial‐temporal reasoning: towards a neurophysiological basis. Neurosci Lett. 1995;185(1):44–7. 7731551 10.1016/0304-3940(94)11221-4

[14] Hughes JR , Daaboul Y , Fino JJ , Shaw GL . The" Mozart effect" on epileptiform activity. Clinical EEG. 1998;29(3):109–19. 10.1177/155005949802900301 9660010

[15] Lin L‐C , Lee W‐T , Wu H‐C , Tsai C‐L , Wei R‐C , Jong Y‐J , et al. Mozart K.448 and epileptiform discharges: effect of ratio of lower to higher harmonics. Epilepsy Res. 2010;89(2–3):238–45. 20129759 10.1016/j.eplepsyres.2010.01.007

[16] Lin LC , Lee MW , Wei RC , Mok HK , Wu HC , Tsai CL , et al. Mozart K.545 mimics Mozart K.448 in reducing epileptiform discharges in epileptic children. Evid Based Complement Alternat Med. 2012;2012(6447):1–6. 10.1155/2012/607517 PMC3523174 23304207

[17] Coppola G , Toro A , Operto FF , Ferrarioli G , Pisano S , Viggiano A , et al. Mozart’s music in children with drug‐refractory epileptic encephalopathies. Epilepsy Behav. 2015;50:18–22. 26093514 10.1016/j.yebeh.2015.05.038

[18] D’Alessandro P , Giuglietti M , Baglioni A , Verdolini N , Murgia N , Piccirilli M , et al. Effects of music on seizure frequency in institutionalized subjects with severe/profound intellectual disability and drug‐resistant epilepsy. Psychiatr Danub. 2017;29:399–404. 28953798

[19] Brackney DE , Brooks JL . Complementary and alternative medicine: the Mozart effect on childhood epilepsy—a systematic review. J School Nurs. 2018;34(1):28–37. 10.1177/1059840517740940 29157096

[20] Maguire M . Epilepsy and music: practical notes. Pract Neurol. 2017;17(2):86–95. 27903764 10.1136/practneurol-2016-001487

[21] Lin LC , Lee MW , Wei RC , Mok HK , Yang RC . Mozart K.448 listening decreased seizure recurrence and epileptiform discharges in children with first unprovoked seizures: a randomized controlled study. BMC Complement Altern Med. 2014;14(1):17. 24410973 10.1186/1472-6882-14-17 PMC3893543

[22] Mansouri A , Fallah A , Valiante TA . . Determining surgical candidacy in temporal lobe epilepsy. Epilepsy Res Treat. 2012;2012:1–16. 10.1155/2012/706917 PMC3420473 22957238

[23] Liégeois‐Chauvel C , Peretz I , Babaï M , Laguitton V , Chauvel P . Contribution of different cortical areas in the temporal lobes to music processing. Brain. 1998;121(10):1853–67. 9798742 10.1093/brain/121.10.1853

[24] Samson S , Zatorre RJ . Learning and retention of melodic and verbal information after unilateral temporal lobectomy. Neuropsychologia. 1992;30(9):815–26. 1407496 10.1016/0028-3932(92)90085-z

[25] Zatorre RJ , Halpern AR . Effect of unilateral temporal‐lobe excision on perception and imagery of songs. Neuropsychologia. 1993;31(3):221–32. 8492875 10.1016/0028-3932(93)90086-f

[26] Johnsrude IS , Owen AM , White NM , Zhao WV , Bohbot V . Impaired preference conditioning after anterior temporal lobe resection in humans. J Neurosci. 2000;20(7):2649–56. 10729345 10.1523/JNEUROSCI.20-07-02649.2000 PMC6772234

[27] Hughes JR , Fino JJ . The Mozart effect: distinctive aspects of the music—a clue to brain coding? Clin Electroencephalogr. 2000;31(2):94–103. 10840632 10.1177/155005940003100208

[28] Theiler J , Eubank S , Longtin A , Galdrikian B , Farmer JD . Testing for nonlinearity in time series: the method of surrogate data. Physica D. 1992;58(1–4):77–94.

[29] Filipan K , Bockstael A , De Coensel B , Schönwiesner M , Botteldooren D . A novel auditory saliency prediction model based on spectrotemporal modulations In: 22nd International Congress on Acoustics (ICA 2016), 2016.

[30] Li T , Yu T , Hawkins BS , Dickersin K . Design, analysis, and reporting of crossover trials for inclusion in a meta‐analysis. PLoS ONE. 2015;10(8):e0133023. 26284684 10.1371/journal.pone.0133023 PMC4540315

[31] Gardner MJ , Altman DG . Confidence intervals rather than P values: estimation rather than hypothesis testing. Br Med J (Clin Res Ed). 1986;292(6522):746–50. 10.1136/bmj.292.6522.746 PMC1339793 3082422

[32] Ho J , Tumkaya T , Aryal S , Choi H , Claridge‐Chang A . Moving beyond P values: everyday data analysis with estimation plots [Internet]. BioRxiv. 2019; 377978. 10.1038/s41592-019-0470-3 31217592

[33] Afra P , Bruggers CS , Sweney M , Fagatele L , Alavi F , Greenwald M , et al. Mobile software as a medical device (SaMD) for the treatment of epilepsy: development of digital therapeutics comprising behavioral and music‐based interventions for neurological disorders. Front Hum Neurosci. 2018;12:171. 29780310 10.3389/fnhum.2018.00171 PMC5946004

[34] Lin LC , Lee WT , Wang CH , Chen HL , Wu HC , Tsai CL , et al. Mozart K. 448 acts as a potential add‐on therapy in children with refractory epilepsy. Epilepsy Behav. 2011;20(3):490–3. 21292560 10.1016/j.yebeh.2010.12.044

[35] Dastgheib SS , Layegh P , Sadeghi R , Foroughipur M , Shoeibi A , Gorji A . The effects of Mozart’s music on interictal activity in epileptic patients: systematic review and meta‐analysis of the literature. Curr Neurol Neurosci Rep. 2013;14(1):420–11. 10.1007/s11910-013-0420-x 24272274

[36] Liao H , Jiang G , Wang X . Music therapy as a non‐pharmacological treatment for epilepsy. Expert Rev Neurother. 2015;15(9):993–1003. 26196169 10.1586/14737175.2015.1071191

[37] Senn S . Cross‐over Trials in Clinical Research. 2nd edn Chichester, Eng.; New York: J. Wiley; 2002:345 (Statistics in practice).

[38] Wellek S , Blettner M . On the proper use of the crossover design in clinical trials. Dtsch Arztebl Int. 2012;109(15):276–81. 22567063 10.3238/arztebl.2012.0276 PMC3345345

[39] Mandell J Tonedeaf Test [Internet]. http://jakemandell.com/tonedeaf/ [cited 2019 Feb 28].

