# Supplementary material for: Daily listening to Mozart reduces seizures in individuals with epilepsy: A randomized control study
Source: Epilepsia Open. 2020 May 27;5(2):285–94. doi: 10.1002/epi4.12400 (PMC7278546; doi:10.1002/epi4.12400)
Supplement: Supplementary file 1 — Supplementary Material [file EPI4-5-285-s001.zip › epi412400-sup-0006-TableS1.docx]

| **Study visit** | **Time** | **Information and data discussed/obtained** |
| --- | --- | --- |
| - **Introductory study visit** | Before starting the baseline period | 1. Pitch perception test 2. Experiencing distress, agitation, tachycardia and rapid breathing building up to a seizure by listening to an specific sound/music previously – Musicogenic Epilepsy 3. Listening previously to the Mozart K. 448 piece, having a memory associated with it and able to recognize the piece in case of exposure. 4. Discussion on how to prepare a seizure diary 5. Discussion on how the participants classify their seizures with the description they provide in their own words, providing guidance on how to mark them separately on their seizure diary. |
| - **1^st^ study visit** | Before 3 months of the baseline period | 1. 20 min EEG recording at rest* |
| - **2^nd^ study visit** | After 3 months of baseline period | 1. 20 min EEG recording at rest* 2. EEG recording while listening to the treatment stimulus for individuals in group A / or listening to the control stimulus for individuals in group B for the first time* 3. Obtaining the seizure diary for the baseline period from the participant |
| - **3^rd^ study visit** | After 3 months of daily listening to either the treatments or control piece depending on the randomization process | 1. 20 min EEG recording at rest* 2. EEG recording while listening to the control stimulus* 3. EEG recording while listening to the treatment stimulus* 4. Obtaining the seizure diary for the past three months |
| - **4^th^ study visit** | After 3 months of daily listening to either the treatments or control piece depending on the randomization process | 1. 20 min EEG recording at rest* 2. EEG recording while listening to the control stimulus for individuals in group A / or listening to the treatment stimulus for individuals in group B for the last time* 3. EEG recording while listening to the treatment stimulus* 4. Obtaining the seizure diary for the past three months |
| - **5^th^ study visit** | After 3 months of the follow-up period | 1. 20 min EEG recording at rest* 2. Obtaining the seizure diary for the past three months |

Table S1- Study visits and description of the collected data during the intervention

**The findings of the EEG recordings will be presented in the future seperatly.*
