# Supplementary figures and images for: Daily listening to Mozart reduces seizures in individuals with epilepsy: A randomized control study
Source: Epilepsia Open. 2020 May 27;5(2):285–94. doi: 10.1002/epi4.12400 (PMC7278546; doi:10.1002/epi4.12400)

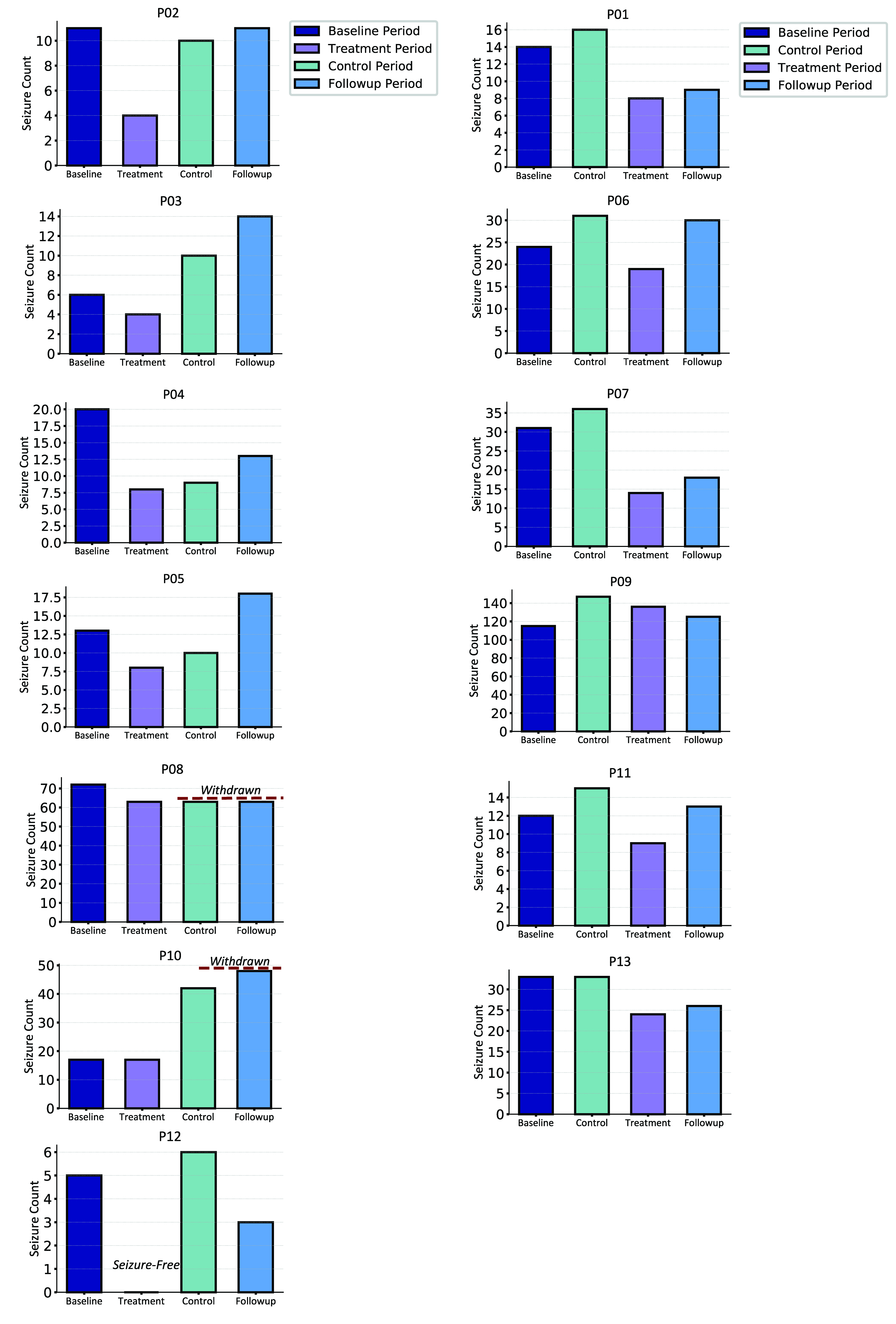

Supplement: Supplementary file 1 — Supplementary Material [file EPI4-5-285-s001.zip › epi412400-sup-0001-FigS1.tif]

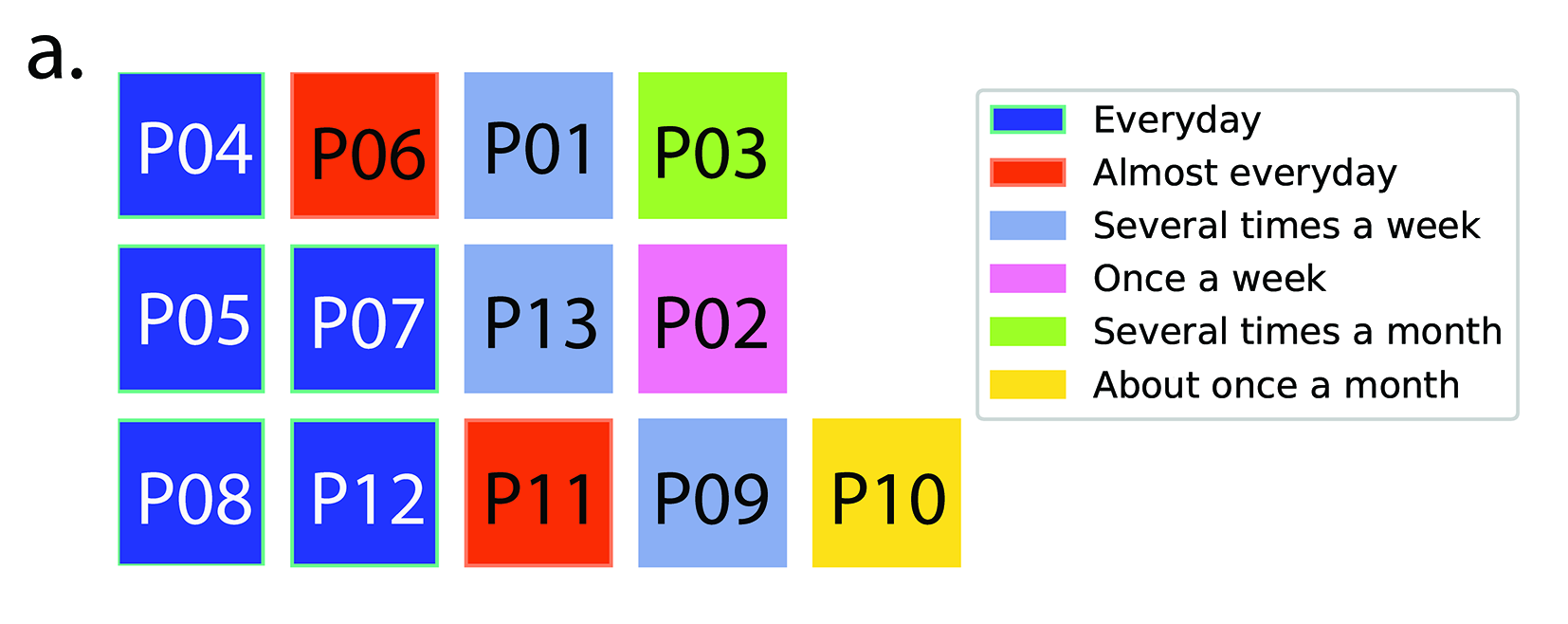

Supplement: Supplementary file 1 — Supplementary Material [file EPI4-5-285-s001.zip › epi412400-sup-0002-FigS2a.tif]

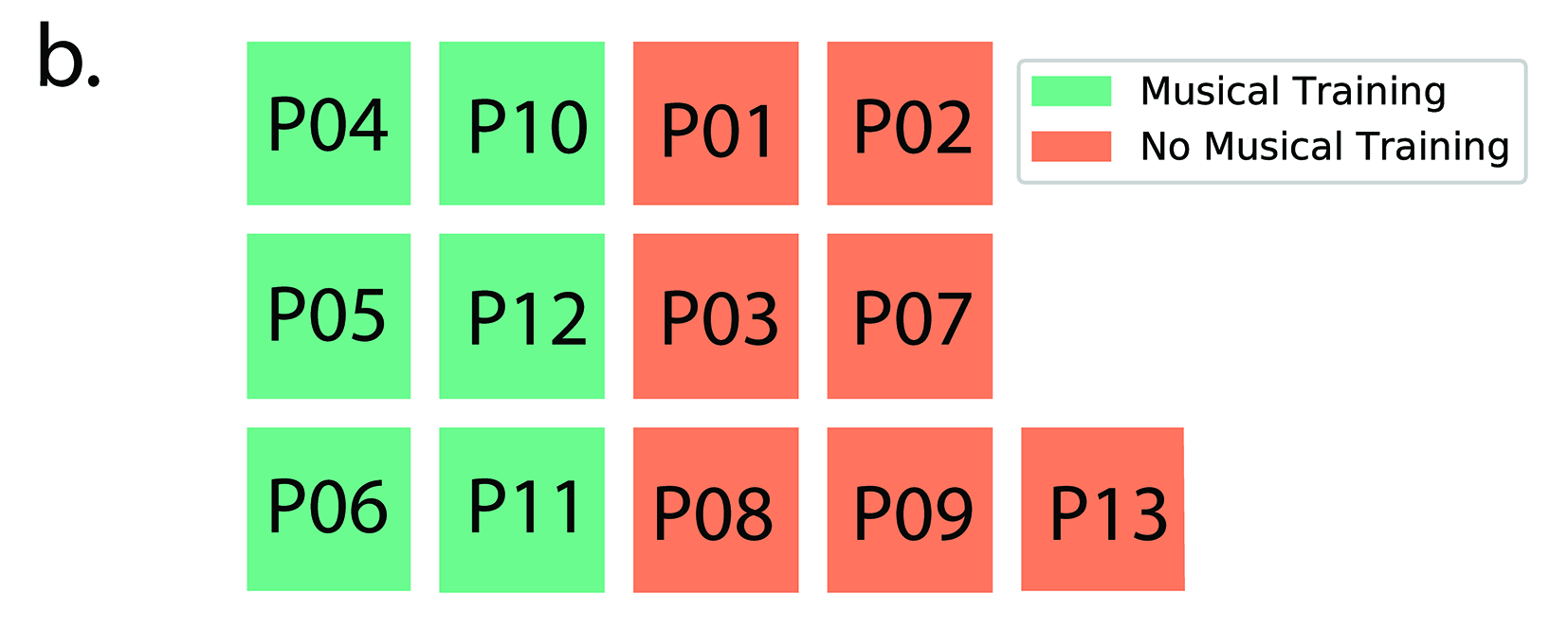

Supplement: Supplementary file 1 — Supplementary Material [file EPI4-5-285-s001.zip › epi412400-sup-0003-FigS2b.tif]

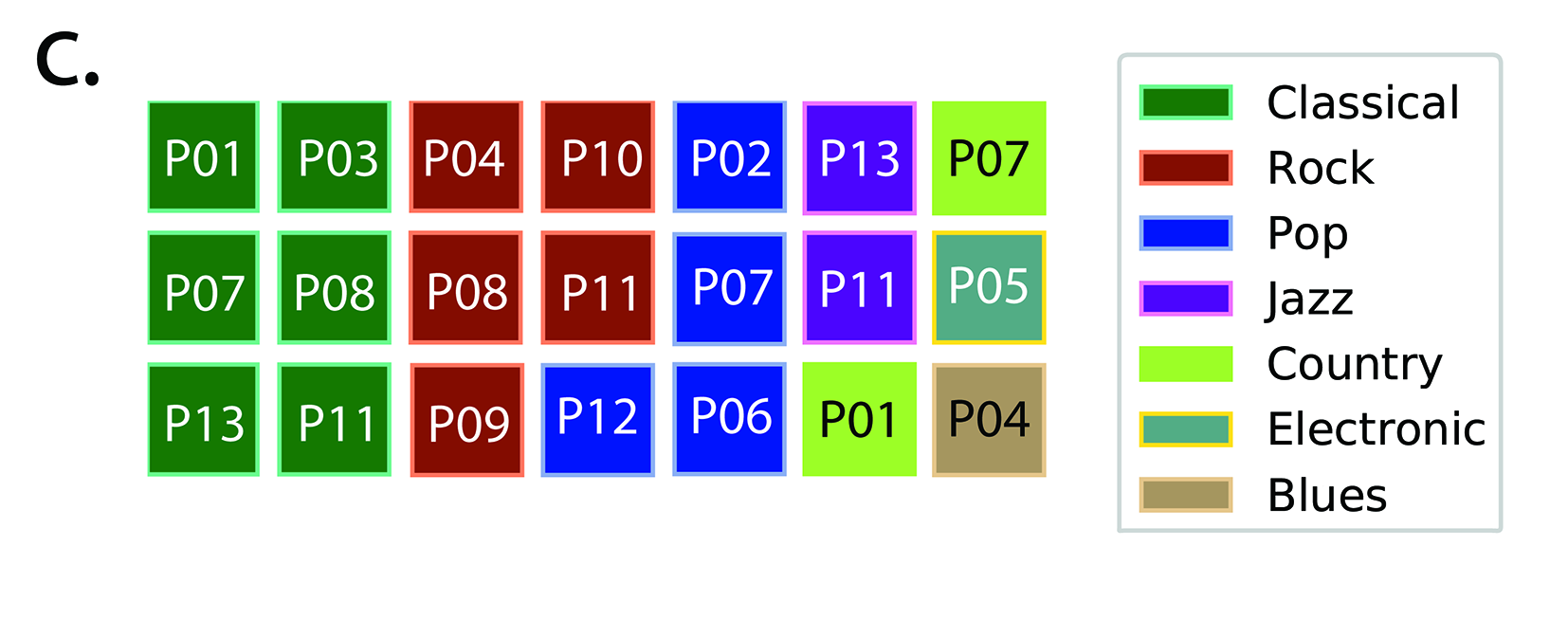

Supplement: Supplementary file 1 — Supplementary Material [file EPI4-5-285-s001.zip › epi412400-sup-0004-FigS2c.tif]

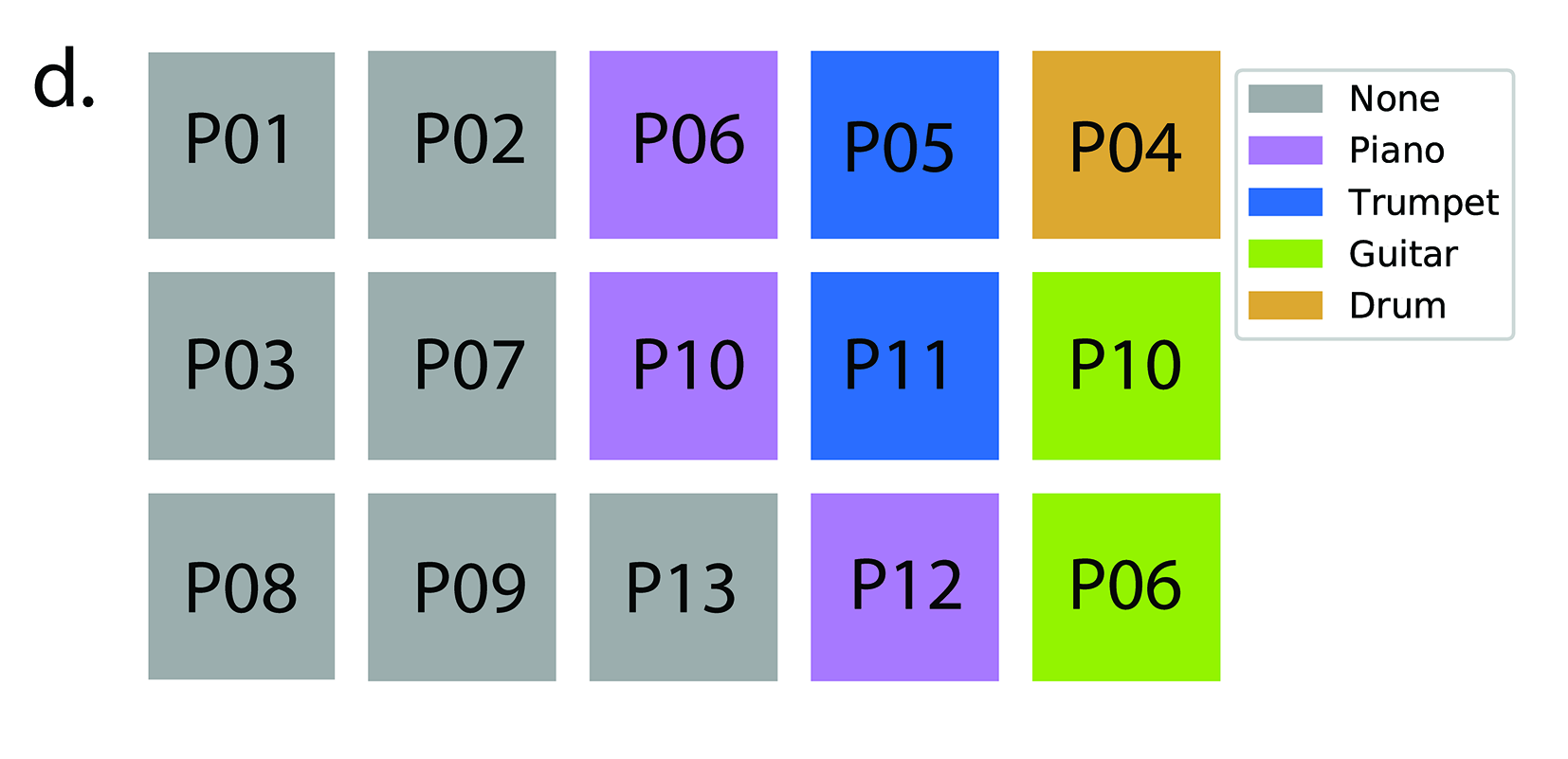

Supplement: Supplementary file 1 — Supplementary Material [file EPI4-5-285-s001.zip › epi412400-sup-0005-FigS2d.tif]
